# Mineral Waters

**Published:** 1867-12

**Authors:** 


### MINERAL WATERS.

"This is like the water of twenty-five years ago," said an habitue of Saratoga Springs the other day as he quaffed his glass at the fountain of Lawrence & Co's Excelsior Water.

It is a well known fact that the powers of the Saratoga waters have diminished greatly of late years in consequence of the fountains having been tampered with, in order to promote a more copious flow of the "health-giving liquids." By a very ingenious device these gentlemen present to those who frequent the springs as well as those who use the water at their own homes, the pure, unadulterated article as it is found in its natural state fifty feet beneath the surface of the soil; it is at that distance the bottles are filled and corked, without any possibility of admixture with the external air or the escapement of their essential virtues; these waters are furnished in their natural state, hence with all their natural virtues. Beyond all question, the best water for a man to drink, in health or disease, is that which is the purest as it comes from its natural fountain. Whatever healthful effects result from the drinking of any impregnated waters is in proportion to the increased action given to the bowels, the kidneys and the skin; by acting on the intestines as mineral salt waters do they expel the excrements of digestion, that is the solid refuse of the food, after the nutritious portions have been extracted and applied to the purposes of sustenance and life. Sulphur waters are known to exert a decided influence on the skin, relaxes it, opens its pores, and allows the passing out of the system a large amount of its impurities, especially the useless portions of the products of combustion of vegetable food in particular, but the waste products of the combustion of animal food are carried away through the kidneys and such waters as act upon them are the most useful in this class of cases, and physicians as well as individuals should remember that the value of all mineral waters depend upon their applicability, in these three forms, and should act accordingly.

